# Peroxisome Proliferator‐Activated Receptor‐γ in Capillary Endothelia Promotes Fatty Acid Uptake by Heart During Long‐Term Fasting

**DOI:** 10.1161/JAHA.112.004861

**Published:** 2013-02-22

**Authors:** Kosaku Goto, Tatsuya Iso, Hirofumi Hanaoka, Aiko Yamaguchi, Toshihiro Suga, Akinari Hattori, Yasunori Irie, Yuji Shinagawa, Hiroki Matsui, Mas Rizky A. A. Syamsunarno, Miki Matsui, Anwarul Haque, Masashi Arai, Fumio Kunimoto, Tomoyuki Yokoyama, Keigo Endo, Frank J. Gonzalez, Masahiko Kurabayashi

**Affiliations:** 1 Department of Medicine and Biological Science, Gunma University Graduate School of Health Sciences, 3‐39‐22 Showa‐machi, Maebashi, Gunma, Japan (K.G., T.I., T.S., H.M., M.R.A.S., M.M., M.A., M.K.); 2 Education and Research Support Center, Gunma University Graduate School of Health Sciences, 3‐39‐22 Showa‐machi, Maebashi, Gunma, Japan (T.I., M.K.); 3 Department of Bioimaging Information Analysis, Gunma University Graduate School of Health Sciences, 3‐39‐22 Showa‐machi, Maebashi, Gunma, Japan (H.H., A.Y.); 4 Department of Laboratory Sciences, Gunma University Graduate School of Health Sciences, 3‐39‐22 Showa‐machi Maebashi, Gunma, Japan (A.H., Y.I., Y.S., H.M., T.Y.); 5 Department of Anesthesiology and Intensive Care Medicine, Gunma University Graduate School of Medicine, Gunma University Graduate School of Health Sciences, 3‐39‐22 Showa‐machi, Maebashi, Gunma, Japan (A.H., F.K.); 6 Department of Diagnostic Radiology and Nuclear Medicine, Gunma University Graduate School of Health Sciences, 3‐39‐22 Showa‐machi, Maebashi, Gunma, Japan (K.E.); 7 Laboratory of Metabolism, Center for Cancer Research, National Cancer Institute, National Institute of Health, Bethesda, MD, USA (F.J.G.)

**Keywords:** capillaries, cardiac metabolism, endothelium, fatty acids, transcription factors

## Abstract

**Background:**

Endothelium is a crucial blood–tissue interface controlling energy supply according to organ needs. We investigated whether peroxisome proliferator‐activated receptor‐γ (PPARγ) induces expression of fatty acid–binding protein 4 (FABP4) and fatty acid translocase (FAT)/CD36 in capillary endothelial cells (ECs) to promote FA transport into the heart.

**Methods and Results:**

Expression of FABP4 and CD36 was induced by the PPARγ agonist pioglitazone in human cardiac microvessel ECs (HCMECs), but not in human umbilical vein ECs. Real‐time PCR and immunohistochemistry of the heart tissue of control (*Pparg*^fl/null^) mice showed an increase in expression of FABP4 and CD36 in capillary ECs by either pioglitazone treatment or 48 hours of fasting, and these effects were not found in mice deficient in endothelial PPARγ (*Pparg*^∆^^EC^^/null^). Luciferase reporter constructs of the *Fabp4* and *CD36* promoters were markedly activated by pioglitazone in HCMECs through canonical PPAR‐responsive elements. Activation of PPARγ facilitated FA uptake by HCMECs, which was partially inhibited by knockdown of either FABP4 or CD36. Uptake of an FA analogue, ^125^I‐BMIPP, was significantly reduced in heart, red skeletal muscle, and adipose tissue in *Pparg*^∆^^EC^^/null^ mice as compared with *Pparg*^fl/null^ mice after olive oil loading, whereas those values were comparable between *Pparg*^fl/null^ and *Pparg*^∆^^EC^^/null^ null mice on standard chow and a high‐fat diet. Furthermore, *Pparg*^∆^^EC^^/null^ mice displayed slower triglyceride clearance after olive oil loading.

**Conclusions:**

These findings identified a novel role for capillary endothelial PPARγ as a regulator of FA handing in FA‐metabolizing organs including the heart in the postprandial state after long‐term fasting.

## Introduction

Endothelium is a crucial blood–tissue interface controlling energy supply according to organ needs. Obesity‐related metabolic disorders such as type 2 diabetes and metabolic syndrome cause endothelial dysfunction. Emerging evidence indicates that endothelial cells (ECs) play an important role in fatty acid (FA) transport from the blood into fat‐utilizing tissues such as heart, red skeletal muscle, and adipose tissue. Because shuttling through the endothelial layer is the first rate‐limiting step in the utilization of long‐chain FAs as fuels, the mechanism of their endothelial transport in heart and skeletal muscle, which contain continuous, nonfenestrated endothelium,^1–2^ has been the subject of intense research. Although several pathways including interendothelial passive diffusion, transcytosis (combination of uptake by endocytosis and discharge by exocytosis) and a facilitated protein‐mediated process have been proposed,^3–4^ the precise mechanisms by which FAs are taken up by muscle tissues are not well understood.

Cytoplasmic fatty acid–binding proteins (FABPs) are a family of 14‐ to 15‐kDa proteins that bind with high affinity to hydrophobic molecules such as long‐chain FAs and eicosanoids.^5^ As lipid chaperones, FABPs may actively facilitate the transport of lipids to specific compartments in the cells, such as to lipid droplets for storage; to the endoplasmic reticulum for signaling, trafficking, and membrane synthesis; and to mitochondria or peroxisomes for oxidation. FABP4, also known as aP2/ALBP/A‐FABP, is expressed highly in adipocytes and much less in macrophages.^6^ Accordingly, the molecular mechanisms regulating FABP4 expression have been extensively studied in adipocytes and macrophages. FABP4 is expressed in the capillary ECs in mouse and human hearts,^7–8^ but it remains to be determined whether FABP4 expression is regulated in ECs by mechanisms similar to those in adipocytes and macrophages. More importantly, the fundamental question of whether endothelial FABP4 contributes to vascular FA transport into heart, skeletal muscle, and adipose tissue has yet to be determined.

Evidence obtained from isolated cells indicates that fatty acid translocase (FAT)/CD36 plays an important role in membrane transport of long‐chain FA uptake in heart and skeletal muscle as well as adipose tissue.^9–10^ Like FABP4, CD36 is also expressed in microvascular ECs,^11–13^ thus suggesting that CD36 is involved in FA transport across the endothelium. Mice lacking CD36 had reduced FA uptake in the heart, skeletal muscle, and adipose tissue, whereas glucose uptake was markedly induced in heart and skeletal muscle, presumably to compensate for the resultant shortage of FA supply.^14–15^ The CD36‐deficient mice also showed an insulin‐tolerant phenotype with increased levels of nonesterified FAs (NEFAs) and triacylglycerol (TG) under a standard chow diet,^16–17^ thus suggesting impaired FA utilization and reciprocally enhanced glucose consumption. Human studies with CD36 mutations showed findings similar to those in the CD36‐deficient mice. Whereas the impact of CD36 mutation on metabolic phenotype is variable in human subjects, myocardial uptake of long‐chain FAs was markedly reduced with enhanced myocardial glucose use in patients with CD36 mutations.^18–20^ However, the relative contribution of endothelial and myocardial CD36 to myocardial FA uptake is not known.

Systemic and cellular lipid metabolism is regulated by peroxisome proliferator‐activated receptors (PPARs): PPARα, PPARβ/δ, and PPARγ. Among these isoforms, PPARγ is primarily a regulator of lipid storage and transport in adipocytes and macrophages, where its constitutive expression is high.^21^ PPARγ2 expression is mainly limited to adipose tissue, whereas PPARγ1 is expressed in various tissues including vascular ECs.^22–23^ Transcriptional activity of PPARγ is modulated by direct binding of small molecules such as long‐chain FAs and synthetic PPARγ agonists such as thiazolidinediones (TZDs), which are clinically utilized as insulin sensitizers.^21,24^ Although induction of FABP4 and CD36 expression by PPARγ agonists was reported in adipocytes and macrophages, regulation of PPARγ‐target genes in the ECs has not been extensively studied.

In the present study, the possibility that PPARγ facilitates FA transport by direct induction of FABP4 and CD36 gene expression in capillary ECs was investigated. By using mice deficient in PPARγ in endothelial cells, evidence is provided that endothelial PPARγ increases FA uptake in heart, skeletal muscle, and adipose tissue under conditions in which the oral lipid load is rapidly increased. Disturbance of transendothelial FA transport regulated by PPARγ results in a remarkable increase in serum levels of TG and NEFAs after olive oil loading, at least partly because of impaired FA uptake by peripheral organs such as heart, red skeletal muscle, and adipose tissue. Thus, capillary endothelial PPARγ is activated during fasting, resulting in more efficient FA transport into FAs utilizing organs such as heart, red skeletal muscle, and adipose tissue when a meal is taken.

## Methods

### Cell Culture

Human cardiac microvessel ECs (HCMECs) were purchased from Lonza (Switzerland) and cultured on collagen‐coated dishes with EBM‐2 medium (Lonza). Human umbilical vein ECs (HUVECs) were obtained from Cell Systems (USA) and cultured on collagen‐coated dishes with CS‐C Complete Medium (Cell Systems).

### Mice

**Figure 1.**
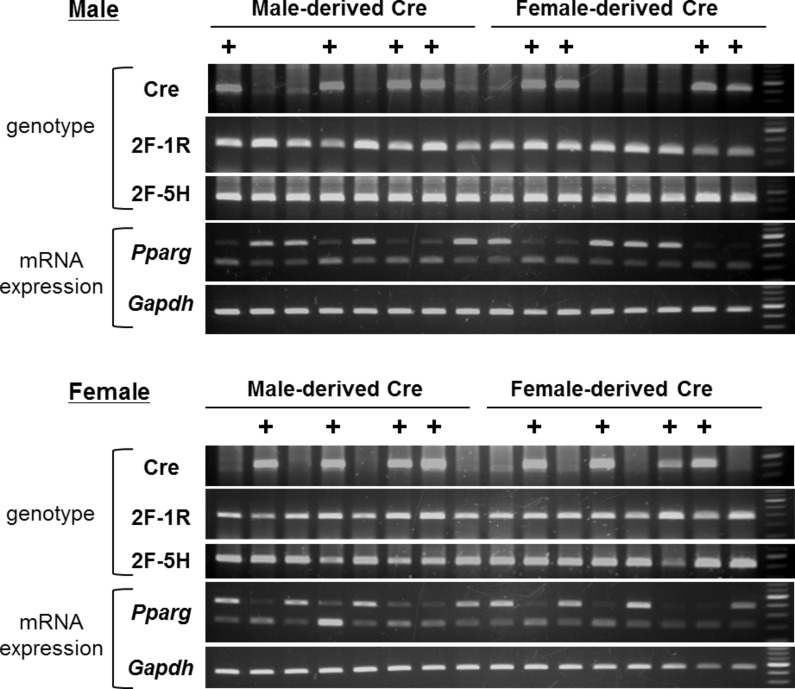
Basal genotype of the mice for this study is *Pparg*^fl/null^, which turn *Pparg*^ΔE/null^ when Cre‐recombinase is expressed in ECs. The presence or absence of Cre‐recombinase and the genotype of *Pparg* were evaluated by PCR. The PCR product by 2F‐1R primers (285 bp) is the floxed allele, whereas the product by 2F‐5H (450 bp) is the null allele.^26^ Detection of both PCR products in a mouse implies that the mouse is *Pparg*^fl/null^. All mice showed the *Pparg*^fl/null^ genotype irrespective of Cre expression. Expression of mRNA for *Pparg* in hearts was also determined by using 1F‐3R primers that can detect both wild‐type (353 bp) and null (214 bp) transcripts. As reported by de Lange, even mice without the Cre‐transgene (Cre‐negative mice) showed modest expression of the null transcript.^26^ However, expression of the null transcript was higher in Cre‐positive mice than in Cre‐negative mice. Importantly, expression of the wild‐type transcript was higher in Cre‐negative mice than in Cre‐positive mice. Taken together, we conclude that the basal genotype of our mice is *Pparg*^fl/null^ and that the mice turn *Pparg*^ΔE/null^ when Cre‐recombinase is expressed in ECs. EC indicates endothelial cell; PPAR, peroxisome proliferator‐activated receptor; PCR, polymerase chain reaction.

Male and female PPARγ‐floxed mice with or without Cre‐recombinase driven by the Tie2 promoter were generated as described previously.^25^ These mice are of mixed C57BL6/N, Sv129, FVB/N background. The genotype of the mice was *Pparg*^fl/null^, and the floxed allele was successfully disrupted when Cre‐recombinase was induced by the Tie2 promoter (Figure 1).^26^ All mice were housed on a 12‐hour light/dark cycle. Before the study, all mice were fed a standard pellet diet (CE‐2, Clea Japan, Inc). Obesity was induced using a high‐fat diet for 12 to 16 weeks, beginning at 6 weeks of age (High Fat Diet 32, Clea Japan, Inc). Low‐fat diet‐fed mice received the standard pellet diet throughout life. Pioglitazone (Takeda Pharmaceutical, Co Ltd) was administered to mice by oral gavage for 14 days (25 mg/kg per day). Animal care and experimentation were approved by the Gunma University Animal Care and Use Committee.

### RNA Isolation and Reverse‐Transcription Polymerase Chain Reaction (RT‐PCR)

Total RNA was isolated from cultured cells and isolated hearts using TRIzol Reagent (Invitrogen). Semiquantitative RT‐PCR was performed with an RT‐PCR kit (TAKARA, Japan) according to the manufacturer's protocol. The gene‐specific primers for cDNA are listed on Table 1. Quantitative real‐time PCR was performed with SYBR Green PCR Master Mix (Applied Biosystems) according to the manufacturer's instructions. Expression of the target gene was normalized to the *Gapdh* mRNA level.

**Table 1.** Primers for RT‐PCR

	Forward	Reverse
hACS	CCAGAAGGGCTTCAAGACTG	TTTGGGGTTGCCTGTAGTTC
hCPT1A	AGTTGGCGTCTGAGAAGCAT	ACACACCATAGCCGTCATCA
hCPT1B	GCAAAGGCCTCATCAAGAAG	CACCTCAGCAAGGAAAGGAG
hCPT2	TTGACTGCGTCCAGTTTCAG	CATTGCTGCTTCTTTGGTCA
hGPIHBP1	ACACAGCAGGAGGAAGAGGA	AATGAGGGTTGTGCAGGTCT
hFABP4	GCTTCCTTCTCACCTTGAAGAA	CCCACAGAATGTTGTAGAGTTC
hFABP5	GGACAGCAAAGGCTTTGATG	GCTCATTGAACTGAGCTTGG
hFAT/CD36	ATGTAACCCAGGACGCTGAG	GTCGCAGTGACTTTCCCAAT
hFATP1	TTGCCTCTGCCTTGATCTTT	TGTCTCCCAGCTGACATGAG
hFATP3	ATACCTGGGAGCGTTTTGTG	GGTCCCCAGTGTTGAAGAAA
hFATP4	CGAGGAGAAGCTGTGATTCC	GTCTGGGGACTTCTGCTCTG
hLPL	GTCCGTGGCTACCTGTCATT	TGGATCGAGGCCAGTAATTC
hPPARγ	AAGGCCATTTTCTCAAACGA	GATGCAGGCTCCACTTTGAT
mFABP4	AAGAAGTGGGAGTGGGCTTT	TCGACTTTCCATCCCACTTC
mFAT/CD36	TGCTGGAGCTGTTATTGGTG	TCTTTGATGTGCAAAACCCA
mPPARγ	CCCTGGCAAAGCATTTGTAT	AATCCTTGGCCCTCTGAGAT
GAPDH	ACCACAGTCCATGCCATCAC	TCCACCACCCTGTTGCTGTA

RT‐PCR indicates reverse‐transcriptase polymerase chain reaction; ACS, acyl CoA synthetase; CPT, carnitine palmitoyltransferase; GPIHBP, glycosylphosphatidylinositol‐anchored high‐density lipoprotein‐binding protein; FABP, fatty acid binding protein; FAT, fatty acid translocase; FATP, fatty acid transport protein; LPL, lipoprotein lipase; PPAR γ, peroxisome proliferator activated receptor‐γ.

### Western Blot Analysis

Western blot analyses were carried out as previously described.^27^ Antibodies against FABP4,^8^ CD36 (Abcam), and GAPDH (Chemicon) were used.

### Immunohistochemical Analysis

Hearts of mice were fixed with 4% paraformaldehyde and embedded in paraffin. Immunohistochemistry was performed with antibody directed against FABP4 using an ABC kit (Vector) according to the manufacturer's protocol. Nuclei were stained with hematoxylin. For immunofluorescence, the cells were labeled with anti‐FABP4 and cy3‐conjugated anti‐rabbit IgG antibody (Sigma).

### Small Interfering RNA (siRNA)

The target sequences of siRNA for FABP4, CD36, PPARγ, GFP, and Lamin were 5′‐AGU UUA UGA GAG AGC AUA AGC ‐3′, 5′‐GAA AUG AUC UUA CUC AGU GUU‐3′, 5′‐GUA CCA AAG UGC AAU CAA AGU‐3′, 5′‐GUU CAG CGU GUC CGG CGA GTT‐3′, and 5′‐CUG GAC UUC CAG AAG AAC ATT‐3′, respectively (Hayashi Kasei). HCMECs were transfected with 20 μmol/L of siRNA by lipofectamine RNAi Max according to the manufacturer's protocol.

### Reporter Gene Assays

The *Fabp4* (aP2) promoter (bp 1‐5491) was a generous gift from Dr. Bruce Spiegelman. The Gateway system (Invitrogen) was used to generate *Fabp4* promoter‐luciferase reporter constructs in adenovirus. A pDONR vector was constructed with multiple cloning sites from the pBluescript (pDONR‐MCS) vector. The SV40 promoter plus the luciferase coding region was subcloned into the pDONR‐MCS (pDONR‐SV40‐luc). The following *Fabp4* promoter regions were introduced into the upstream region of the SV40 promoter of pDONR‐SV40‐luc by PCR: bp 1 to 140, 1 to 240, 1 to 929, 121 to 240, 221 to 929, 862 to 2300, 2130 to 3896, and 3171 to 5491. To generate the mutated *Fabp4* reporter constructs, 2 consensus PPARγ‐binding sites (adipocyte regulatory elements; ARE6 and ARE7) in the *Fabp4* promoter (bp 1 to 240) were changed by PCR (ARE6; ACATTTCACC to mutated ARE6; CCCCCGGGGG, ARE7; GATCAGAGTT to mutated ARE7; CCCCCGGGGG). Adenovirus‐reporter constructs with these *Fabp4* promoters were produced by using the Gateway system as described previously.^27^ The *CD36* V1/V3 promoter (1510 bp) was a generous gift from Dr Kiyoto Motojima (P2 reporter plasmid).^28^ The following V1/V3 promoter fragments were subcloned upstream of the SV40 promoter of pDONR‐SV40‐luc by PCR: bp 1 to 1510, 1 to 730, 710 to 1510, 710 to 1150, and 1131 to 1510. To generate the mutated CD36 reporter construct, a PPARγ‐responsive element (PPRE) in the V1/V3 promoter (bp 710 to 1150) was changed by PCR (original PPRE; TGGCCTCTGACTT to mutated PPRE; ACCTAAGCTTGAA). We also isolated the V2 (2466 bp) and V4/V5 (2351 bp) promoters from the human genome by PCR using primers 5′‐ACATGGGAAGTGCTGGGTAG‐3′ and 5′‐GAAATGAGGCACAGGCTCTC‐3′ for V2 and 5′‐AGGGCAGGGAAAGCTATTGT‐3′ and 5′‐CGTATCATTTTGCCCGTTCT‐3′ for V4/V5 and subcloned them into pDONR‐SV40‐luc. *CD36* promoter‐luciferase reporter constructs of the adenovirus were generated as described above. The cells were infected with adenovirus‐reporter constructs at an m.o.i. of 20. Luciferase assays were performed at least twice using a luciferase assay system (Promega).

### Fatty Acid Uptake

For the fatty acid uptake experiments, HCMECs were starved in DMEM without glucose and bovine fetal serum for 30 minutes. Ten minutes after adding a mixture of ^14^C‐palmitic acid (Perkin Elmer, USA) plus bovine serum albumin, cells were washed with ice‐cold stop buffer (PBS containing 0.1% bovine serum albumin and 0.2 mmol/L phloretin) and lysed with lysis buffer (0.1*N* NaOH and 0.2% SDS). Radioactivity of the lysate in the scintillation cocktail (Aquasol2; Perkin Elmer) was measured by a liquid scintillation counter (LCS‐3000; Aloka). Experiments were done in triplicate and repeated 3 times.

### Biodistribution of ^125^I‐BMIPP and ^18^F‐FDG

Biodistribution of 15‐[p‐iodophenyl]‐3‐[R,S]‐methyl pentadecanoic acid (^125^I‐BMIPP) and 2‐fluorodeoxyglucose (^18^F‐FDG) was determined as described previously.^14–15^ Mice received intravenous injections of ^125^I‐BMIPP (5 kBq) and ^18^F‐FDG (100 kBq) via the lateral tail vein in a volume of 100 μL. ^125^I‐BMIPP was a gift from Nihon Medi‐Physics Co Ltd, and ^18^F‐FDG was obtained from batches prepared for clinical PET imaging at Gunma University. The animals were euthanized 2 hours after injection. The isolated tissues were weighed and counted in a well‐type gamma counter (ARC‐7001; Aloka). Each experiment was performed at least twice.

### Measurement of TG, NEFAs, Glucose, and Insulin

Serum glucose was measured by a Glutest sensor (Sanwa Kagaku; Aichi, Japan). Serum levels of insulin (Mercodia; Uppsala, Sweden), triglyceride (Triglyceride E‐test; Wako Chemical, Osaka), nonesterified fatty acid (NEFA C‐test; Wako Chemical, Osaka, Japan) and ketone bodies (BioAssay Systems, CA) were measured according to the manufacturers' protocols.

### Statistical Analysis

Statistical analysis was performed in SPSS 20.0. Data are presented as dot plots or mean±SD. Statistical comparisons were performed using nonparametric analysis (Mann–Whitney *U* test) when the group numbers were 2. Statistical significance was tested by the Kruskal–Wallis test with the Bonferroni post hoc test when experiments included >3 groups. The level of significance was set at a probability value of <0.05. Effect size (ES) was shown with η^2^ and ω^2^.

## Results

### PPARγ Regulated Expression of FABP4 and CD36 in Capillary ECs In Vitro

**Figure 2.**
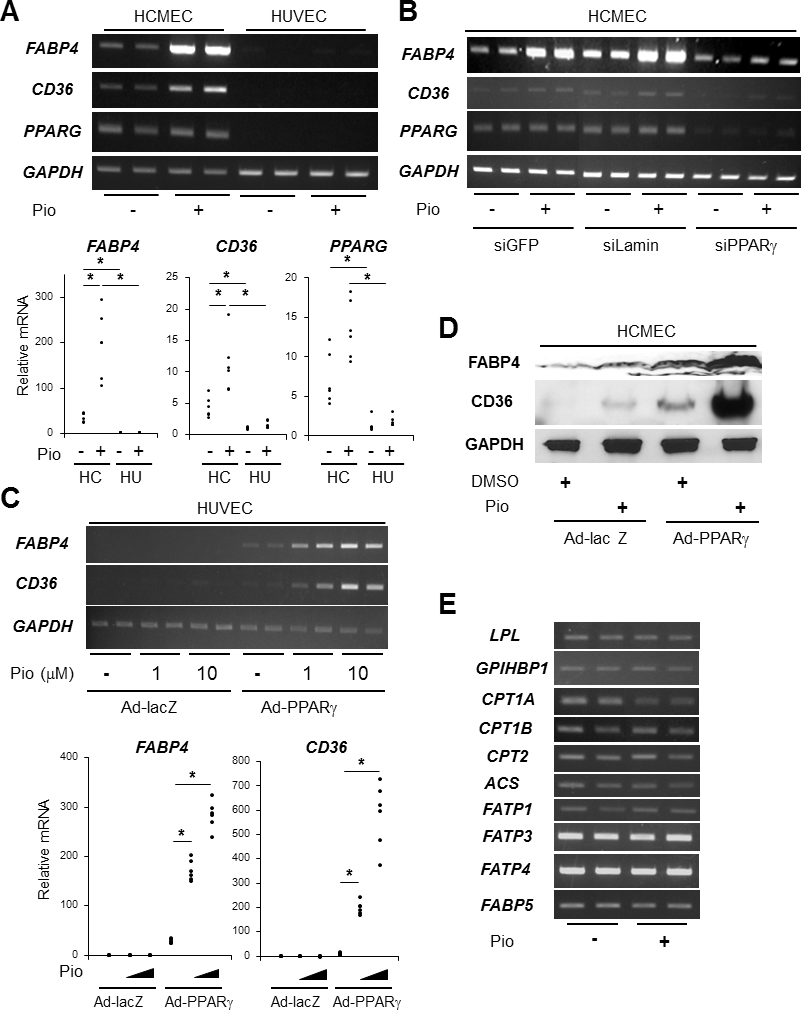
Effects of PPARγ agonist and PPARγ knockdown and overexpression on FABP4 and CD36 expression in ECs in vitro. A, Human cardiac microvessel ECs (HCMECs, HC) and human umbilical vein ECs (HUVECs, HU) were treated with pioglitazone (Pio, 10 μmol/L) or DMSO. Two days later, total RNA was extracted for PCR (n=6 in each group); *P<0.05. Effect size (ES): η^2^=0.85, ω^2^=0.82 for *FABP4*; η^2^=0.75, ω^2^=0.70 for *CD36*; and η^2^=0.82, ω^2^=0.79 for *PPARG*. B, After pretreatement with siGFP, siLamin, or siPPARγ, HCMECs were treated with pioglitazone (10 μmol/L). Two days later, total RNA was isolated for PCR. C, HUVECs were infected with Ad‐lacZ or Ad‐PPARγ at an m.o.i. of 20 in the presence or absence of pioglitazone (10 μmol/L). Two days later, total RNA was extracted for PCR (n=6 in each group); *P<0.05. ES: η^2^=0.99, ω^2^=0.99 for *FABP4*; η^2^=0.92, ω^2^=0.91 for *CD36*. D, HCMECs were infected with Ad‐lac Z or Ad‐PPARγ at an m.o.i. of 20 in the presence or the absence of pioglitazone treatment. Three days later, protein was extracted for Western blot analysis. GAPDH was used as an internal control. E, HCMECs were treated with pioglitazone (10 μmol/L) or DMSO. Two days later, total RNA was extracted for RT‐PCR. Expression of indicated genes was determined. LPL functions as triglyceride hydrolase for chyromicrons and very‐low‐density lipoprotein to liberate NEFAs at the surface of capillary ECs. GPIHBP1 is expressed in capillary ECs and transports LPL across ECs to the capillary lumen. CPT1a, CPT1b, and CPT2 are the key enzymes in carnitine‐dependent transport across the mitochondrial membrane and rate‐limiting factors for FA β‐oxidation. CPT1a is predominantly expressed in liver but is also expressed in ECs. ACS converts NEFAs into fatty acyl**‐**CoA esters in cytoplasm. FATP1, FATP3, FATP4 are involved in translocation of long‐chain fatty acids across the plasma membrane. FATP3 and FATP4 are induced in capillary ECs by vascular endothelial growth factor–B. FABP5, also known as epidermal FABP and mal1, is a member of the FABP family and is strongly expressed in capillary ECs. Note that these molecules are *not* induced by pioglitazone. PPAR indicates peroxisome proliferator‐activated receptor; EC, endothelial cell; PCR, polymerase chain reaction; RT, reverse transcription; LPL, lipoprotein lipase; GPIHBP1, glycosylphosphatidylinositol‐anchored high‐density lipoprotein‐binding protein; CPT, carnitine palmitoyltransferase; ACS, acyl CoA synthetase; NEFA, nonesterified fatty acid; FATP, fatty acid transport protein; FABP, fatty acid–binding protein; DMSO, dimethyl sulfoxide; DMEM, Dulbecco's modified eagle's medium.

To explore the role of PPARγ in ECs, the effects of pioglitazone, a PPARγ ligand, was examined in HCMECs and HUVECs. Expression of *FABP4* and *CD36* mRNA was induced by pioglitazone in HCMECs that express PPARγ, but not in HUVECs with no detectable *PPARγ* mRNA (Figure 2A). When HCMECs were pretreated with siRNA for PPARγ, the increase in *FABP4* and *CD36* mRNA levels by pioglitazone was completely abolished (Figure 2B). Adenoviral overexpression of PPARγ in HUVECs and HCMECs increased pioglitazone‐induced expression of *FABP4* and *CD36* mRNA (Figure 2C) and protein (Figure 2D). In contrast, mRNA encoding lipoprotein lipase (LPL), glycosylphosphatidylinositol‐anchored high‐density lipoprotein‐binding protein (GPIHBP1), CPT1/2, ACS, FATP1/3/4, and FABP5 was not increased by pioglitazone (Figure 2E). These results suggest that the genes encoding FABP4 and CD36 are most highly responsive to PPARγ.

### PPARγ Regulated Expression of FABP4 and CD36 in Capillary ECs In Vivo

**Figure 3.**
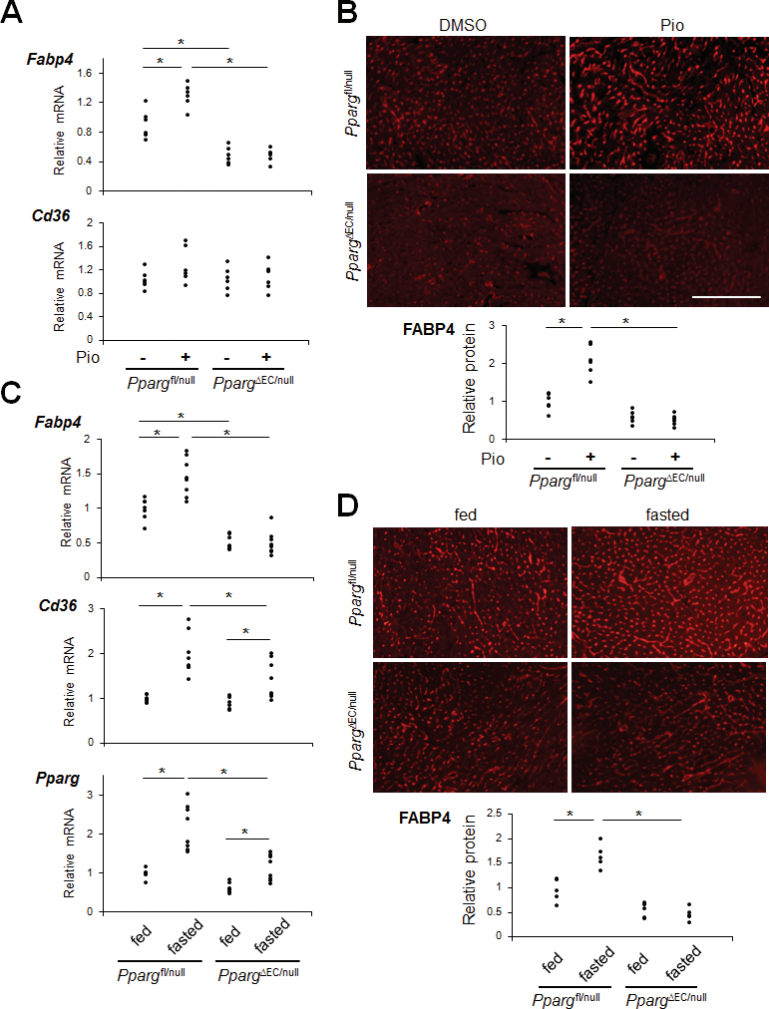
Effects of pioglitazone and fasting on FABP4 and CD36 expression in *Pparg*^fl/null^ and *Pparg*^∆EC/null^ mice hearts. A, *Pparg*^fl/null^ and *Pparg*^∆EC/null^ mice were treated with pioglitazone (25 mg/kg per day) or vehicle for 2 weeks. Total RNA was extracted from hearts for qPCR analysis. *Gapdh *mRNA was used as an internal control (n=7 to 9 in each group), *P<0.05. ES: η^2^=0.86, ω^2^=0.84 for *Fabp4*; η^2^=0.07, ω^2^=−0.07 for *Cd36*. B, Immunofluorescence of FABP4 in hearts of *Pparg*^fl/null^ and *Pparg*^∆EC/null^ mice with or without pioglitazone treatment (25 mg/kg per day) for 2 weeks. Scale bar: 200 μm. Intensity of FAPB4‐positive area was calculated as previously described using ImageJ software^29^ (National Institutes of Health); n=6 in each group; *P<0.05. C, Total RNA was extracted from heart tissues of *Pparg*^fl/null^ and *Pparg*^∆EC/null^ mice for qPCR after 0, 24, or 48 hours of fasting (n=7 to 9 in each group); *P<0.05. ES: η^2^=0.84, w^2^=0.81 for *Fabp4*; η^2^=0.66, ω^2^=0.61 for *Cd36*; η^2^=0.76, ω^2^=0.72 for *Pparg*. D, Immunofluorescence of FABP4 in *Pparg*^fl/null^ and *Pparg*^∆EC/null^ mice hearts with or without 24 hours' fasting. Scale bar: 200 μm. Intensity of FAPB4‐positive area was calculated using ImageJ software (n=6 in each group); *P<0.05. PPAR indicates peroxisome proliferator‐activated receptor; EC, endothelial cell; ES, effect size; FABP, fatty acid binding protein; qPCR, quantitative polymerase chain reaction.

We next examined whether the *Fabp4* and *Cd36* genes are targets of PPARγ in vivo by using PPARγ endothelial null (*Pparg*^∆EC/null^) mice.^25^ When mice were treated with pioglitazone, expression of *Fabp4* mRNA was induced in control mice (*Pparg*^fl/null^), but not in the *Pparg*^∆EC/null^ mice (Figure 3A). In contrast, *Cd36* mRNA was not induced by pioglitazone. Enhancement of *Fabp4* expression by pioglitazone was observed in capillary ECs in the *Pparg*^fl/null^ mice (Figure 3B).^29^ These findings demonstrate that *Fabp4* was induced by pioglitazone in capillary ECs, not in other cell types, in a PPARγ‐dependent manner. We next examined whether the expression of FA‐handing genes is induced by fasting given that PPARγ is activated by fasting. Results of quantitative real‐time PCR showed that mRNA encoding *Fabp4* and *Cd36* as well as *Pparg* was increased after 24 hours of fasting in the *Pparg*^fl/null^ mice (Figure 3C). Among these, induction of *Fabp4* expression was completely abolished in *Pparg*^∆EC/null^ mice, whereas *Cd36* and *Pparg* expression remained inducible although to a lesser extent in the *Pparg*^∆EC/null^ mice (Figure 3C). These data suggest that both CD36 and PPARγ are expressed in ECs as well as other cell types including cardiomyocytes and that lack of PPARγ in ECs leads to a slight reduction in expression of PPARγ in whole hearts of the *Pparg*^∆EC/null^ mice, resulting in a slight reduction in CD36 expression. Increased immunoreactivity against FABP4 was exclusively observed in capillary ECs in *Pparg*^fl/null^ mice (Figure 3D), thus indicating that capillary‐specific expression of FABP4 is enhanced by fasting in a PPARγ‐dependent manner.

**Figure 4.**
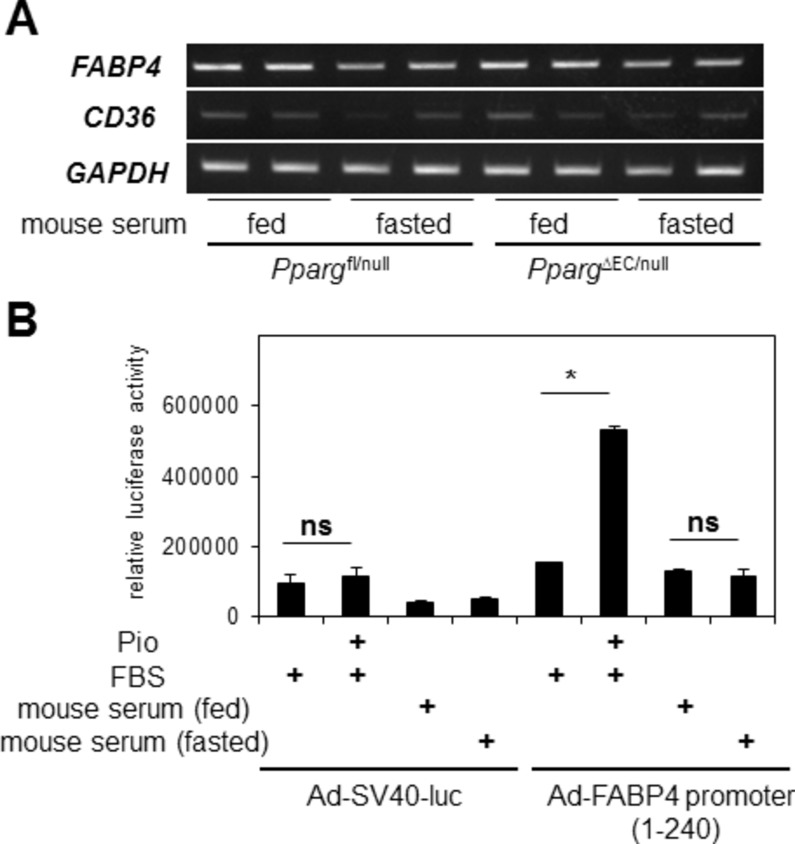
Serum from fasted mice did not induce expression of FABP4 and CD36 in HCMECs. A, Serum was collected from *Pparg*^fl/null^ and *Pparg*^∆^^EC^^/null^ mice with or without 48‐hour starvation. HCMECs were treated with 10% of the serum for 3 days. Total RNA was extracted for PCR. B, HCMECs were infected with the reporter‐adenovirus containing the *Fabp4* promoter (bp 1 to 240) at an m.o.i. of 20 in the presence of serum described above. Fetal bovine serum (FBS) plus pioglitazone (10 μmol/L) was used as a positive control. Three days later, cells were lysed for the luciferase assay (n=4); *P<0.05. PPAR indicates peroxisome proliferator‐activated receptor; HCMEC, human cardiac microvessel endothelial cells; PCR, polymerase chain reaction; FABP, fatty acid binding protein.

To determine whether the induction of FABP4 and CD36 after fasting depends on serum components changed by fasting, HCMECs were cultured in medium supplemented with 10% mouse serum derived from fed or 48‐hour fasted mice. There was no significant difference in their expression between the groups (Figure 4A). Transcriptional activity of the *Fabp4* promoter (described below in detail) was not enhanced by the serum either (Figure 4B), suggesting that serum alone is not sufficient to induce expression of FABP4 and CD36 mRNA.

### FABP4 Promoter Was Transactivated Via 2 Canonical PPREs in Capillary ECs

**Figure 5.**
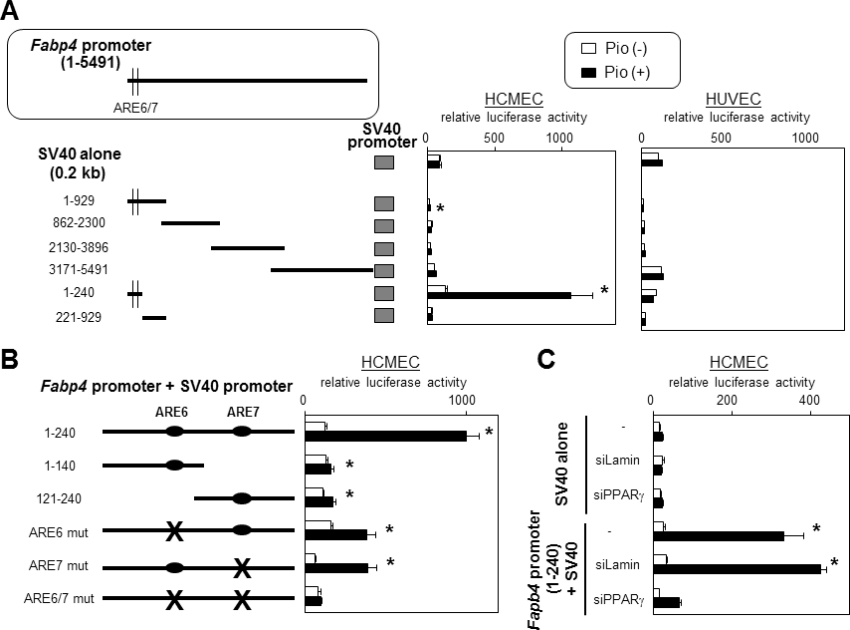
Determination of pioglitazone‐responsive elements within FABP4 promoter. A, HCMECs and HUVECs were infected with reporter‐adenoviruses containing the indicated constructs of *Fabp4* promoter at an m.o.i. of 20 in the presence of pioglitazone (Pio, 10 μmol/L) or DMSO. Three days later, cells were lysed for the luciferase assay (n=4 in each group); *P<0.05. B, HCMECs were infected with reporter‐adenoviruses containing the indicated constructs of the *Fabp4* promoter at an m.o.i. of 20 in the presence of pioglitazone (10 μmol/L) or DMSO (n=4 in each group); *P<0.05. C, After pretreatment with siLamin or siPPARγ, HCMECs were infected with reporter‐adenovirus containing the *Fabp4* promoter (bp 1 to 240) in the presence of pioglitazone (10 μmol/L) or DMSO (n=4 in each group); *P<0.05. FABP indicates fatty acid binding protein; HCMEC, human cardiac microvessel endothelial cells; HUVEC, human umbilical vein endothelial cells; ARE, adipocyte responsive element; DMSO, dimethyl sulfoxide.

To examine the transcriptional regulation of the *Fabp4* promoter in ECs, adenovirus‐reporter constructs were generated because standard lipofection severely damaged ECs, resulting in poor transfection efficiency. The *Fabp4* promoter (bp 1 to 5491) was divided into 6 fragments: bp 1 to 929, 1 to 240, 221 to 929, 862 to 2300, 2130 to 3896, and 3171 to 5491. Pioglitazone induced transcriptional activity of *Fabp4* promoters containing 2 canonical PPREs (bp 1 to 929 and 1 to 240) in HCMECs, but not in HUVECs (Figure 5A). Further analysis utilizing the bp 1 to 240 fragment revealed that both canonical PPREs are required for transactivation of the *Fabp4* promoter (Figure 5B). Knockdown of PPARγ expression by siRNA abolished pioglitazone‐induced *Fabp4* expression (Figure 5C). These findings lent further support to the hypothesis that pioglitazone induces FABP4 expression by PPARγ in capillary ECs where PPARγ is expressed.

### CD36 Promoter Was Transactivated via a PPRE in Capillary ECs

**Figure 6.**
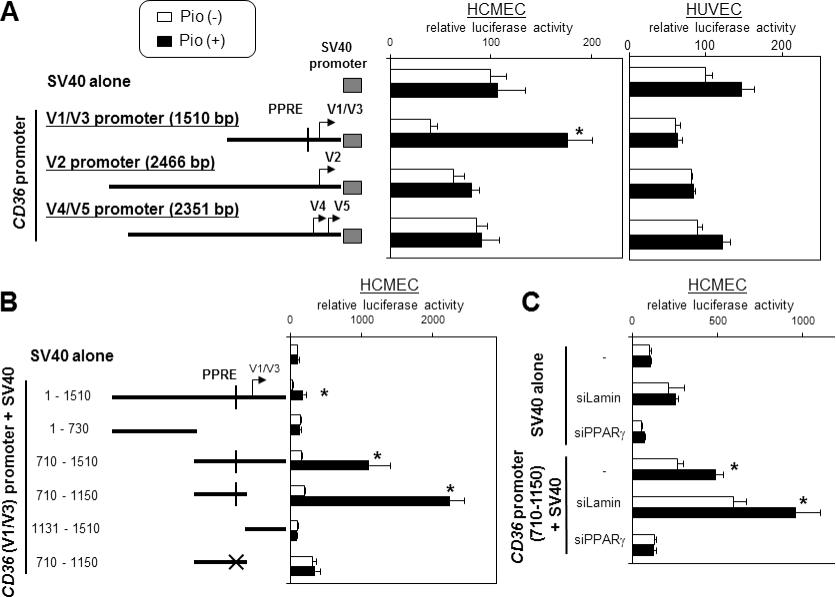
Determination of pioglitazone‐responsive elements within *CD36* promoter. A, HCMECs and HUVECs were infected with reporter‐adenoviruses containing the indicated constructs of the *CD36* promoter at an m.o.i. of 20 in the presence of pioglitazone (Pio, 10 μmol/L) or DMSO. Three days later, cells were lysed for the luciferase assay (n=6 in each group); *P<0.05. B, HCMECs were infected with reporter‐adenoviruses containing the indicated constructs of the *CD36* promoter at an m.o.i. of 20 in the presence of pioglitazone (10 μmol/L) or DMSO (n=6 in each group); *P<0.05. C, After pretreatment with siLamin or siPPARγ, HCMECs were infected with reporter‐adenovirus containing the *CD36* promoter (bp 710 to 1510) in the presence of pioglitazone (10 μmol/L) or DMSO (n=6 in each group); *P<0.05. HCMEC indicates human cardiac microvessel endothelial cells; HUVEC, human umbilical vein endothelial cells; PPAR, peroxisome proliferator activated receptor; DMSO, dimethyl sulfoxide.

**Figure 7.**
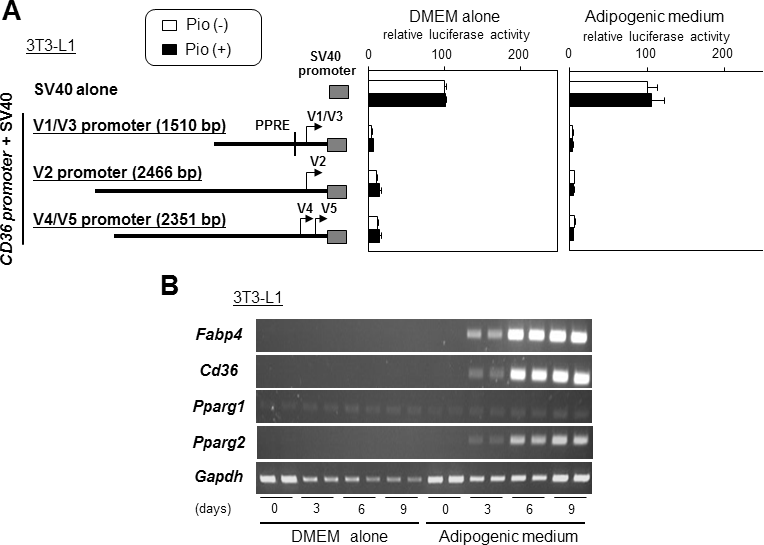
*CD36* promoter did not respond to pioglitazone stimulation in 3T3L1 adipocytes. A, 3T3L1 fibroblasts and adipocytes were infected with reporter‐adenoviruses containing the indicated constructs of the *CD36* promoter at an m.o.i. of 20 with or without adipogenic medium (1 μg/mL insulin, 1 μmol/L dexamethasone, and 500 μmol/L 3‐isobutyl‐1‐methylxantine). Three days later, cells were lysed for the luciferase assay (n=3); *P<0.05. B, 3T3L1 cells were treated with and without adipogenic medium. Total RNA was extracted for RT‐PCR. Expression of the indicated genes was determined. Note that endogenous *Fabp4*,* Cd36*, and *Pparg2* were induced in 3T3L1 adipocytes when treated with adipogenic medium. PPAR indicates peroxisome proliferator‐activated receptor; RT‐PCR, reverse‐transcriptase polymerase chain reaction.

Transcriptional regulation of the *CD36* promoter in ECs was also examined. Because *CD36* mRNA is transcribed from several different promoters, 3 adenovirus‐reporter constructs of *CD36* promoters, termed V1/V3, V2, and V4/V5, were generated. V1/V3 contains a putative PPRE that has questionable biological significance.^28,30^ Transcriptional activity of the V1/V3 promoter was increased by pioglitazone in HCMECs, but not in HUVECs (Figure 6A), whereas activity of the V2 and the V4/V5 promoters was not enhanced in both HCEMCs and HUVECs. Constructs that lacked the PPRE within the V1/V3 promoter showed no responsiveness to pioglitazone (Figure 6B). PPARγ knockdown by siRNA completely abolished pioglitazone‐induced activation of the V1/V3 promoter (Figure 6C). As described previouly,^29^ these promoters were not responsive to PPARγ in 3T3L1 adipocytes despite the abundant expression of *Pparg2* as well as *Cd36* (Figure 7A and 7B). Collectively, these findings indicate that the V1/V3 PPRE mediates pioglitazone‐induced expression of CD36 in capillary ECs and suggest that both FABP4 and CD36 are direct targets of PPARγ in capillary ECs.

### FA Uptake Was Promoted by PPARγ Stimulation Via Induction of FABP4 and CD36

**Figure 8.**
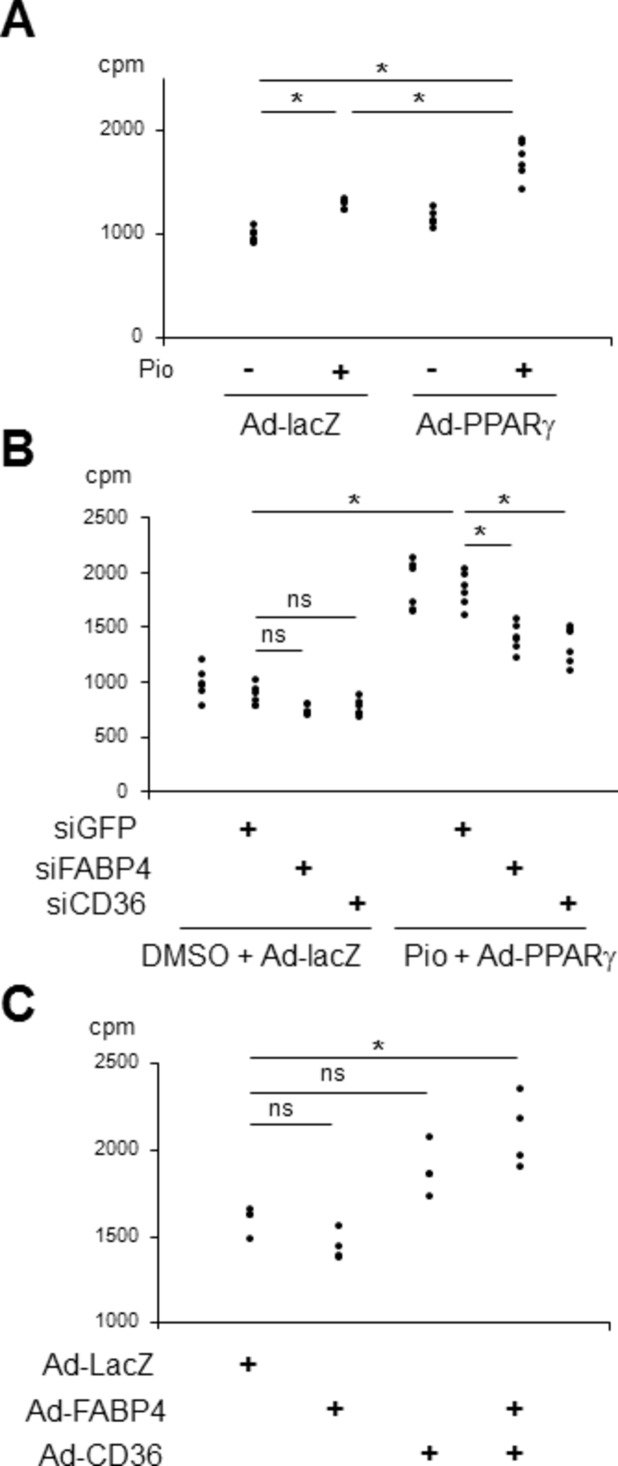
Contribution of FABP4 and CD36 to pioglitazone‐induced FA uptake by HCMECs. A, HCMECs were infected with Ad‐lacZ or Ad‐PPARγ at an m.o.i. of 20 in the presence of pioglitazone (Pio, 1 μmol/L) or DMSO. Forty‐eight hours later, ^14^C‐palmitic acid uptake was measured (n=6 in each group); *P<0.05. ES: η^2^=0.89, ω^2^=0.87. B, After pretreatment with siGFP, siFABP4, or siCD36, HCMECs were infected with Ad‐lacZ or Ad‐PPARγ at an m.o.i. of 20 in the presence of pioglitazone (1 μmol/L). Forty‐eight hours later, ^14^C‐palmitic acid uptake was measured (n=6 in each group); *P<0.05. ES: η^2^=0.92, ω^2^=0.91. C, HCMECs were infected with Ad‐lacZ, Ad‐FABP4, Ad‐CD36, or Ad‐FABP4 plus Ad‐CD36 at an m.o.i. of 10. Forty‐eight hours later, ^14^C‐palmitic acid uptake was measured (n=4 in each group); *P<0.05. ES: η^2^=0.82, ω^2^=0.77. FA, fatty acid; FABP indicates fatty acid binding protein; HCMEC, human cardiac microvessel endothelial cells; PPAR, peroxisome proliferator‐activated receptor; ES, effect size; DMSO, dimethyl sulfoxide.

To determine the role of FABP4 and CD36 in capillary ECs, FA uptake was examined using ^14^C‐palmitic acid. FA uptake was increased by pioglitazone and further enhanced by overexpression of PPARγ (Figure 8A and 8B). Pretreatment of HCMECs with siFABP4 or siCD36 diminished FA uptake induced by pioglitazone plus overexpression of PPARγ, suggesting that FABP4 and CD36 contribute to FA uptake (Figure 8B). Interestingly, FA uptake was increased only when both FABP4 and CD36 were overexpressed, and such an increase in FA uptake was less than that by PPARγ overexpression plus pioglitazone (Figure 8C). These findings suggest that both FABP4 and CD36 play a role in FA uptake, whereas either FABP4 or CD36 alone is not sufficient. Our data also suggest that other target genes of PPARγ are likely to be involved in FA uptake in combination with FABP4 and FAT/CD36.

### FA Uptake Was Impaired in Heart, Red Skeletal Muscle, and Adipose Tissue in Pparg∆EC/null Mice After Olive Oil Gavage

**Figure 9.**
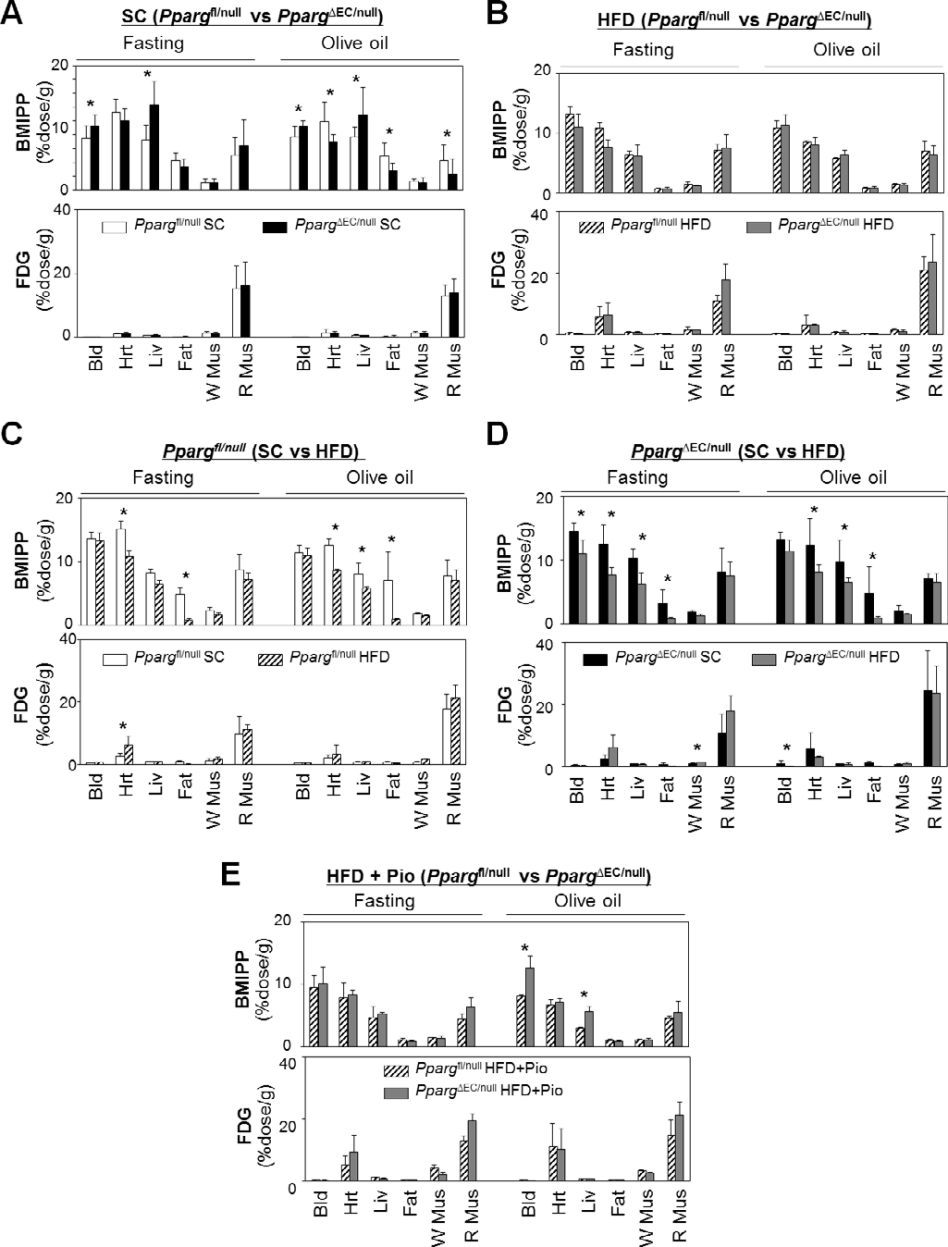
FA uptake by various tissues in *Pparg*^fl/null^ and *Pparg*^∆^^EC^^/null^ mice. Uptake of ^125^I‐BMIPP and ^18^F‐FDG by organs from *Pparg*^fl/null^ and *Pparg*^∆^^EC^^/null^ mice with or without olive oil gavage was measured as described in Methods (n=6 in each group); *P<0.05. A and B, Mice were fed (A) a standard chow (SC) or (B) a high‐fat diet (HFD) and starved for 24 hours before experiments. C and D, Mice were fed SC or HFD and starved for 24 hours before experiments. Uptake of ^125^I‐BMIPP and ^18^F‐FDG by organs from *Pparg*^fl/null^ (C) or *Pparg*^∆^^EC^^/null^ mice (D) was measured. E, Mice were fed with HFD in treatment with pioglitazone (25 mg/kg per day) for 14 days and starved for 24 hours before experiments. FA indicates fatty acid; PPAR, peroxisome proliferator‐activated receptor; ^125^I‐BMIPP, 15‐(p‐iodophenyl)‐3‐(R,S)‐methyl pentadecanoic acid; ^18^F‐FDG, 2‐fluorodeoxyglucose; Bld, blood; Hrt, heart; Liv, liver; Fat, gonadal fat pad; W Mus, white skeletal muscle; R Mus, red skeletal muscle.

To determine the physiological relevance of the regulation of *FABP4* and *CD36* gene expression by PPARγ in ECs, biodistribution of the slowly oxidized FA analogue ^125^I‐BMIPP and metabolically trapped glucose analogue ^18^F‐FDG was compared. Uptake of ^125^I‐BMIPP by heart, adipose tissue, and red skeletal muscle was comparable between standard chow‐fed *Pparg*^fl/null^ and *Pparg*^∆EC/null^ mice after 24 hours of fasting (Figure 9A) and even after refeeding with standard chow (data not shown), whereas uptake of ^125^I‐BMIPP by liver was higher in the *Pparg*^∆EC/null^ mice. We then tested whether endothelial PPARγ disruption interferes with lipid metabolism in the endothelium when serum FA levels are excessively increased. Standard chow‐fed *Pparg*^fl/null^ and *Pparg*^∆EC/null^ mice were subjected to a 24‐hour fasting period followed by olive oil gavage. Interestingly, uptake of ^125^I‐BMIPP was significantly lower in heart, adipose tissue, and red skeletal muscle in the *Pparg*^∆EC/null^ mice (Figure 9A). Uptake of ^125^I‐BMIPP in the liver was higher after olive oil gavage (Figure 9A), which probably reflects a compensatory influx of FAs into the liver. These findings suggest that although endothelial PPARγ is dispensable for FA supply to heart, adipose tissue, and red skeletal muscle via capillary endothelium in the fasting state, endothelial PPARγ is required for the uptake of excess FAs after a lipid‐rich diet following fasting.

Next, the impact of endothelial PPARγ deficiency on FA uptake in the overnutrient state was examined. *Pparg*^fl/null^ and *Pparg*^∆EC/null^ mice fed a high‐fat diet (HFD) for 4 months were subjected to 24 hours of fasting followed by olive oil gavage and measuring the uptake of ^125^I‐BMIPP and ^18^F‐FDG. The *Pparg*^fl/null^ and the *Pparg*^∆EC/null^ mice displayed no significant difference during the fasted and olive oil–fed state (Figure 9B). ^125^I‐BMIPP uptake by heart and adipose tissue was markedly reduced in both *Pparg*^fl/null^ and *Pparg*^∆EC/null^ mice (Figure 9C and 9D). This reduction was also observed after olive oil gavage (Figure 9C and 9D). These results indicate that a deficiency in endothelial PPARγ does not lead to a reduction in FA transport into heart and adipose tissue when mice are fed HFD. This is likely because of the capacity of parenchymal cells for FA uptake that seems to be saturated despite the status of capillary‐endothelial function.

We further studied how pioglitazone affects FA uptake by peripheral organs. When mice were fed HFD, pioglitazone did not alter the uptake of both ^125^I‐BMIPP and ^18^F‐FDG in heart and adipose tissue (Figure 9E). However, uptake of ^125^I‐BMIPP in liver and serum level were lower in the *Pparg*^fl/null^ mice (Figure 9E), suggesting that uptake of ^125^I‐BMIPP via capillary ECs was improved by pioglitazone at the whole‐body level in the *Pparg*^fl/null^ mice. Although the effect of pioglitazone on capillary ECs after HFD is unclear at the individual organ level, it is likely that the gross effect of pioglitazone for metabolism in the whole body is improved via the capillary ECs.

### TG and NEFA Clearance Was Slower in Pparg^∆EC/null^ Than in Pparg^fl/null^ Mice

**Figure 10.**
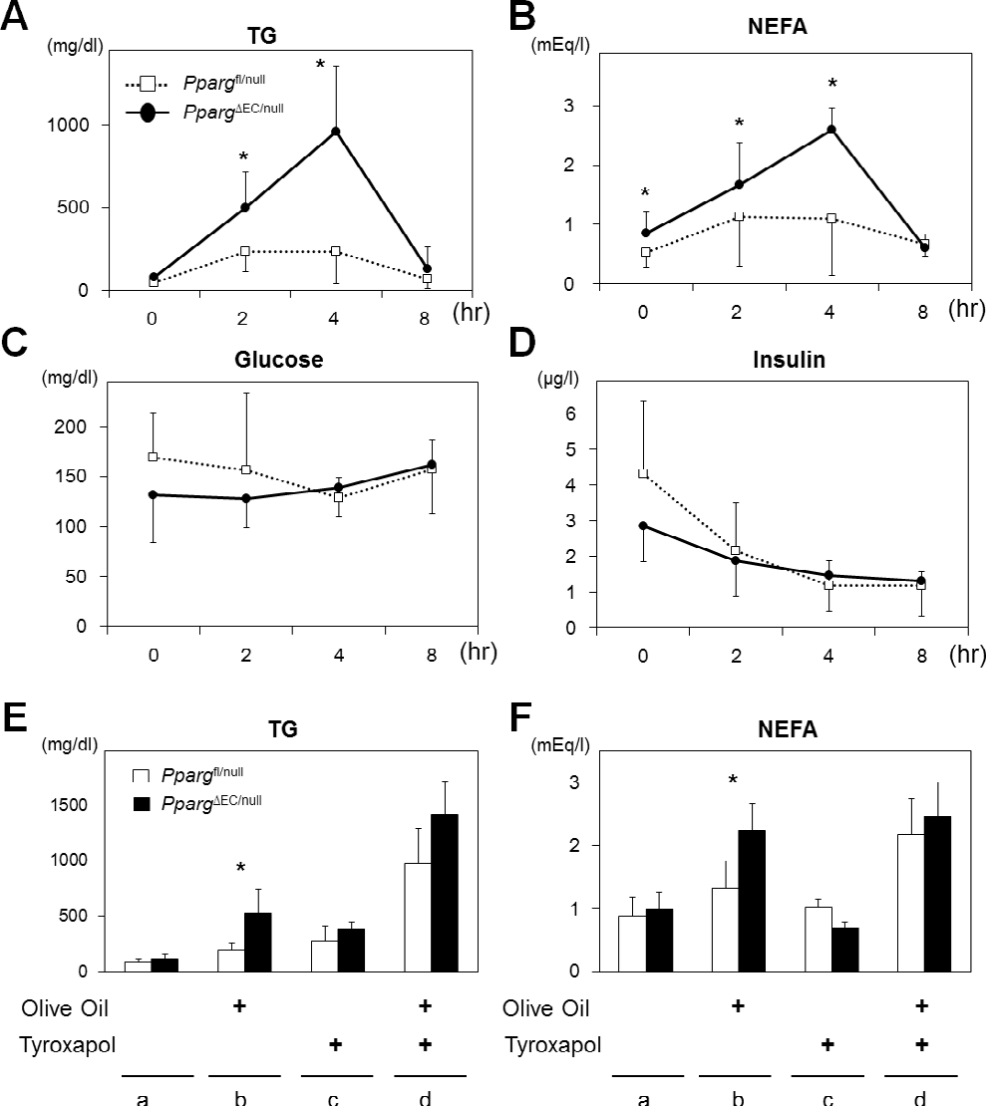
TG and NEFA clearance was disturbed in *Pparg*^∆^^EC^^/null^ mice. A through D, Serum levels of TG, NEFAs, glucose, and insulin in *Pparg*^fl/null^ and *Pparg*^∆^^EC^^/null^ mice were measured at the indicated time after olive oil gavage after 24 hours of fasting (n=6 in each group); *P<0.05. □/dotted line, *Pparg*^fl/null^ mice, ●/solid line, *Pparg*^∆^^EC^^/null^ mice. E and F, Serum levels of TG and NEFAs in *Pparg*^fl/null^ and *Pparg*^∆^^EC^^/null^ mice were measured 2 hours after olive oil gavage after 24 hours of fasting in the presence or absence of treatment with tyroxapol (500 mg/kg); n=6 in each group; *P<0.05. TG indicates triacylglycerol; NEFA, nonesterified fatty acid; PPAR, peroxisome proliferator‐activated receptor;

Serum levels of TG, NEFAs, glucose, insulin, and ketone bodies after olive oil gavage to *Pparg*^∆EC/null^ and *Pparg*^fl/null^ mice were examined. Serum TG (Figure 10A) and NEFAs (Figure 10B) were markedly increased in the *Pparg*^∆EC/null^ mice 2 and 4 hours after olive oil gavage, whereas serum glucose (Figure 10C), insulin (Figure 10D), and ketone bodies (data not shown) were not affected. The influence of hepatic VLDL production plus lipid absorption from the gastrointestinal (GI) tract was determined by using tyloxapol (WR‐1339), an LPL inhibitor (Figure 10E and 10F). *Pparg*^fl/null^ and *Pparg*^∆EC/null^ mice were divided into 4 groups: (1) no treatment, (2) olive oil gavage, (3) tyloxapol treatment (hepatic VLDL production), and (4) tyloxapol treatment plus olive oil gavage (hepatic VLDL production+lipid absorption from GI tract) (Figure 10E and 10F). Although serum levels of TG and NEFAs were higher in the *Pparg*^∆EC/null^ mice after olive oil gavage (2), the difference disappeared on treatment with tyloxapol (4), thus suggesting that the sum of hepatic VLDL production plus lipid absorption from the GI tract is comparable between the *Pparg*^fl/null^ and the *Pparg*^∆EC/null^ mice. Accordingly, the difference in TG and NEFAs levels after olive oil gavage (2) is likely a result of a disturbance in FA uptake by peripheral organs. Taken together, these data suggest that PPARγ in capillary ECs regulates efficient FA uptake by peripheral FAs consuming organs such as heart, red skeletal muscle, and adipose tissue when serum levels of lipids are rapidly increased in the postprandial state.

## Discussion

Despite intense research, the mechanisms underlying FA uptake and transport remain elusive, particularly in heart and skeletal muscle, where energy substrates are supplied through muscle‐type continuous capillaries. Here, by using mice deficient in endothelial PPARγ and systemic administration of ^125^I‐BMIPP, a long‐chain FA analogue that allows the evaluation of FAs in tissues of whole‐body capillary endothelial PPARγ was demonstrated to facilitate FA transport in heart and skeletal muscle through induction of FABP4 and CD36. In addition, this study has provided definitive evidence indicating that ligand activation of PPARγ leads to transcriptional activation of the *FABP4* and *CD36* genes through canonical PPREs in cardiac microvessel ECs. Furthermore, these experiments showed that endothelial disruption of PPARγ increases plasma levels of TG and NEFAs, indicating that ECs play a role in controlling systemic lipid metabolism in the postprandial state. These findings corroborate earlier findings demonstrating that PPARγ deficiency in ECs caused marked dyslipidemia after a high‐fat diet or olive oil gavage.^31^

### Physiological Relevance of PPARγ Regulation of FABP4 and CD36 in Muscle‐Type Capillary ECs

It is well known that PPARγ exerts its control of metabolic activities by increasing white adipose tissue mass, leading to efficient energy conservation and storage by adipocytes along with improved glucose homeostasis. These activities were evolutionally beneficial for mammals to survive food shortage or famine.^24^ Thus, from an evolutionary perspective, PPARγ activation favors survival in an ancient era. In this regard, the present findings indicating PPARγ‐driven induction of FABP4 and CD36 by long‐term fasting are especially noteworthy because this mechanism may represent a novel aspect of the thrifty activities of PPARγ in heart, red skeletal muscle, and adipose tissue. Indeed, cardiac muscle is the most energy‐requiring tissue in the body and primarily uses FAs and, to a great extent, lipoprotein‐derived FA. During long‐term fasting, circulating NEFA levels are elevated by an increase in lipolysis in adipose tissue. Under this condition, activation of PPARγ expression may be adaptive in order for the myocardium to take up FAs vigorously to support ATP synthesis.

When fed an HFD, however, the difference in FA uptake between the *Pparg*^fl/null^ and *Pparg*^∆EC/null^ mice was reduced in all organs tested before as well as after olive oil loading. This is likely because of FA uptake by parenchymal cells of individual organs that was markedly decreased both in the *Pparg*^fl/null^ and the *Pparg*^∆EC/null^ mice to the same degree from longtime lipid overload. This is consistent with a previous report that fat storage of adipose tissue after meals was substantially depressed in obese men.^32^ In our model, the capacity of FA uptake by parenchymal cells (myocytes, adipocytes, and hepatocytes) was already saturated before olive oil loading, resulting in no obvious enhancement of FA uptake. Moreover, when treated with pioglitazone under HFD, a difference in FA transport into heart and adipose tissue did not appear again. Clearance of ^125^I‐BMIPP from circulation, however, was decreased in *Pparg*^fl/null^ mice, suggesting that the sum of a small improvement in capillary function by pioglitazone leads to a significant effect on whole‐body metabolism. Consistent with this finding, it was reported that rosiglitazone significantly lowered NEFA and TG levels in *Pparg*^fl/null^ mice receiving a lipid load, whereas rosiglitazone had no effect on either NEFA or TG levels in the *Pparg*^∆EC/null^ mice.^31^ Thus, impaired transendothelial FA transport by loss of PPARγ in capillary ECs modestly affects diet‐induced dyslipidemia. In this regard, capillary endothelial PPARγ can be a therapeutic target of dyslipidemia induced by a HFD.

### Upstream Signal That Stimulates PPARγ

What are the factors responsible for an induction of PPARγ target genes in the fasting response? Since the initial observation by Amri et al,^33^ there has been increasing evidence that FAs are potent regulators of lipid metabolism. It is well established that PPARs are FA‐responsive transcription factors, and FAs serve as ligands for the 3 PPARs. Although many studies described that FA derivatives of arachidonic acid such as the eicosanoids leukotriene B4, carbaprostacycline, and unsaturated FAs are PPAR activators, most of these ligands were identified by in vitro approaches, and thus the bona fide endogenous PPAR ligands remain to be discovered.^34^ In a recent study, Chakravarthy et al^35^ identified a phosphatidylcholine species, 1‐palmitoyl‐2‐olelyl‐sn‐glycerol‐3‐phosphocholine (16:0/18:1‐GPC), as a physiologically relevant endogenous PPARα ligand. However, no significant difference in the expression of FABP4 and CD36 in HCMECs between the groups on treatment with serum from fed and 48‐hour fasted mice was observed. In addition, no endogenous ligands of PPARγ relevant to the fasting response have emerged to date.

### FABP4 Promoter Was Active in Capillary ECs

In the present study, FABP4 was found to be expressed in capillary ECs, and its promoter strongly activated by PPARγ and pioglitazone via 2 canonical PPREs. These findings are rather surprising because *Fabp4* (also called aP2) has long been considered to be an adipocyte‐ and macrophage‐specific gene, and its promoter has been widely used to generate “fat‐specific” expression and disruption in transgenic mouse studies. In these experiments, “fat‐specific” Cre expression was achieved by placing the Cre cDNA under the control of the 5.4‐kb promoter fragment of the *Fabp4* gene.^36^ Therefore, it is likely that Cre expression is induced in ECs, and the resultant gene of interest is disrupted by recombination in ECs as well as in adipocytes and macrophages. Accordingly, caution should be advised when analyzing and interpreting phenotypes of knockout mice, when said phenotypes may be a result at least partly of the deficiency of the gene of interest in the ECs.

### Differential Regulation of CD36 Promoter Between Adipocyte and Capillary ECs

CD36 is a multifunctional membrane‐glycoprotein expressed in various cells including adipocytes, striated muscle, cardiomyocytes, smooth muscle, microvessel endothelium, platelets, macrophages, and hepatocytes.^9,37^ CD36 has ≥5 spliced variants that are differentially regulated by divergent promoters. Among them, the present study revealed that the V1/V3 promoter is responsible for capillary‐endothelial expression of CD36 in a PPARγ‐dependent manner. This promoter contains a putative PPRE element that was first reported by Tontonoz et al,^30^ who showed transactivation of the minimal length promoter by a synthetic PPARγ ligand and overexpressing exogenous PPARγ1 and RXR in CV‐1 cells (monkey kidney fibroblasts).^30^ In contrast, others reported that mouse and human proximal *CD36* promoters containing a PPRE did not respond to PPARα and PPARγ ligands in rat hepatoma Fao cells and 3T3L1 cells.^28^ However, the present study clearly showed that the PPRE contained in the V1/V3 promoter is functionally important in PPARγ‐dependent expression of CD36 in capillary ECs. On the other hand, these promoters are not responsive to PPARγ in 3T3L1 adipocytes, despite the abundant expression of endogenous *Pparg2* as well as *Cd36*. These results indicate that the V1/V3 promoter of the *CD36* gene is differentially regulated between ECs and adipocyte, and suggest that cofactors that associated with the PPARγ/RXR heterodimer, and/or the interaction between PPARγ/RXR and tissue‐specific transcription factors may play a role in cell‐type‐specific function of PPARγ. Further study would be warranted.

### Involvement of Endothelial PPARγ in Hydrolysis of TG‐Rich Lipoproteins

It is well known that hearts utilize the long‐chain FAs associated with albumin or derived from LPL‐mediated hydrolysis of triglyceride‐rich lipoproteins. Lipase activity of LPL requires a newly recognized partner protein, GPIHBP1, which is expressed exclusively in capillary ECs and transports LPL across ECs to the capillary lumen.^38^ Because TG level was markedly increased after olive oil loading in *Pparg*^ΔE/null^ mice, we assumed that LPL activity is impaired by decreased expression of LPL or GPIHBP1. However, neither *LPL* nor *GPIHBP1* gene expression was induced by pioglitazone in HCMECs, whereas both were induced in both *Pparg*^fl/null^ and *Pparg*^ΔE/null^ mice after 24 hours' fasting (data not shown). Given that PPARγ controls multiple FA‐handling genes, hydrolysis of TG‐rich lipoproteins through the induction of LPL activity may also be regulated by PPARγ through genes or mechanisms yet to be identified.

In conclusion, PPARγ induces FABP4 and CD36 expression in capillary ECs in the heart and plays a role in FA uptake by the heart that heavily utilizes FAs as the main substrates for energy conversion. The present study has revealed a novel role for the PPARγ‐mediated physiological response during severe fasting.
